# Communicating hunger and satiation in the first 2 years of life: a systematic review

**DOI:** 10.1111/mcn.12230

**Published:** 2015-12-01

**Authors:** Janet McNally, Siobhan Hugh‐Jones, Samantha Caton, Carel Vereijken, Hugo Weenen, Marion Hetherington

**Affiliations:** ^1^ Institute of Psychological Sciences University of Leeds Leeds UK; ^2^ School of Health and Related Research (ScHARR) University of Sheffield Sheffield UK; ^3^ Danone Nutricia Research Utrecht Netherlands

**Keywords:** infant feeding, hunger, satiation, behaviour, cue, communication

## Abstract

Responsive feeding has been identified as important in preventing overconsumption by infants. However, this is predicated on an assumption that parents recognise and respond to infant feeding cues. Despite this, relatively little is understood about how infants engage parental feeding responses. Therefore, the aim of this systematic review was to identify what is known about infant communication of hunger and satiation and what issues impact on the expression and perception of these states. A search of Medline, CINAHL, Web of Science, PsycINFO, Science Direct and Maternal and Infant care produced 27 papers. Eligibility criteria included peer reviewed qualitative and/or quantitative publications on feeding behaviours, hunger, and satiation/satiety cues of typically developing children in the first 2 years of life. Papers published between 1966 and 2013 were included in the review. The review revealed that feeding cues and behaviours are shaped by numerous issues, such as infants' physical attributes, individual psychological factors and environmental factors. Meanwhile, infant characteristics, external cues and mothers' own characteristics affect how feeding cues are perceived. The existing literature provides insights into many aspects of hunger and satiation in infancy; however, there are significant gaps in our knowledge. There is a lack of validated tools for measuring hunger and satiation, a need to understand how different infant characteristics impact on feeding behaviour and a need to extricate the respective contributions of infant and maternal characteristics to perceptions of hunger and satiation. Further research is also recommended to differentiate between feeding driven by liking and that driven by hunger.

## Introduction

Childhood obesity is prevalent in developed countries (Ogden et al. 2012; Wang & Lobstein 2006), and research has focussed on factors that might increase obesity risk in children. Some of the factors identified thus far include parental body mass index (BMI), birthweight, early adiposity, weight gain during the first year of life and maternal feeding practices (Dev et al. 2013; Reilly et al. 2005).

Several reviews indicate that maternal feeding practices may increase obesity risk by influencing the early entrainment of appetite control (De Lauzon‐Guillain et al. 2012; Disantis et al. 2011; Hurley et al. 2011). However, the precise mechanisms linking feeding practices and childhood obesity remain unclear. DiSantis et al. (2011) proposed a theoretical role for maternal feeding ‘responsiveness’ in infant and child overweight. ‘Responsive’ mothers are sensitive to hunger and satiation cues and respond to these appropriately, while discordant maternal responses are a proposed risk factor for obesity. Worobey et al. (2009) found lower maternal sensitivity to feeding cues at 6 months predicted infant weight gain between 6 and 12 months. Hurley et al. (2011) also found two types of discordant response, restrictive feeding and indulgent feeding, to be associated with a high BMI in infants and young children. Meanwhile, DiSantis et al. (2011) suggested that a third kind of discordant response, maternal pressure to eat, may also increase obesity risk. Evidence for the latter view is mixed as Farrow & Blissett (2008) found pressure to eat at 1 year to be associated with lower weight at 2 years. However, Farrow & Blissett (2006) found infants with high weight gain in the first 6 months whose mothers exhibited pressure to eat continued on this trajectory between 6 and 12 months. In addition, Lumeng et al. (2012) found assertive prompts to eat and maternal intrusiveness to be associated with higher adiposity in toddlers. Poor responsiveness to satiation cues in the form of pressure to eat may therefore also affect obesity risk.

Notwithstanding reported associations between maternal responsiveness and infant adiposity, the direction of causality between these remains unclear. Overfeeding may arise from insensitivity to fullness cues or the use of food to settle fractious infants (Worobey et al. 2009; Redsell et al. 2010). Restrictive feeding practices may also play a role by increasing the desirability, and consequent consumption, of restricted foods (Dev et al. 2013). Importantly though, mothers may simply be responding to their child's appetite (Webber et al. 2010a) as some infants have a more avid appetite than others (Agras et al. 1990). In turn, mothers may restrict intake for children they perceive to over‐eat or may pressure children with small appetites to eat more (Webber et al. 2010b). There is therefore a need to better understand the issues that affect interpretations of and responses to infant feeding cues in order to develop interventions to prevent overfeeding. The aim of the current review was to consider the evidence regarding what infants communicate during meals and what parents respond to. Specifically, the review aimed to identify the following:

1. How hunger and satiation are expressed in infants and toddlers.
2. The issues that impact on the expression and perception of infant feeding cues.
3. How hunger and satiation behaviour can be differentiated from eating driven by the hedonic features of food.

**Key messages**

- Hunger cues are easier to perceive by mothers than satiation cues, and feeding cues are easier to interpret as children grow older.
- Infant feeding cues are diverse and highly variable across and within individuals being influenced by many issues including age, sex, genotype, developmental maturity and feeding method.
- Feeding interactions are dyadic in nature, and both infant characteristics (age, sex and temperament) and maternal characteristics (e.g. body mass index) may affect how feeding cues are perceived.
- There is a need to develop methods for measuring infant hunger and satiation and for discriminating feeding driven by hunger from that driven by liking. Additional research is also recommended regarding the impact of different infant characteristics on feeding behaviour and of different maternal and infant characteristics on perceptions of hunger and satiation.

## Method

### Search strategy

An initial scoping exercise was conducted to establish whether reviews had been completed previously on infant feeding cues. The Cochrane Systematic Review Database was searched followed by Medline, CINAHL, Web of Science, PsycINFO, Science Direct and Maternal and Infant care. The scoping exercise was also used to generate search terms and synonyms and to establish the utility of the databases for the search. Final keyword search terms appear in Table 1.

**Table 1:** Final search terms

(Infan^*^ OR baby OR babies OR toddler^*^ or newborn^*^ or neonate^*^)
AND
(Feed^*^ OR eat^*^ OR hunger OR satiety OR satiation OR fullness OR meal^*^)
AND
(cue^*^ OR behavio?r or behavio?rs OR sign^*^ OR communication)

Keyword searches were conducted up to January 2014. Where databases offered combined keyword and subject heading search options (Medline, Maternal and Infant Health and PsycINFO), search terms (infant and feeding) were mapped to subject headings. Following keyword and combined keyword and subject heading searches, results were refined by applying initial limiters: English language, full text, peer reviewed, human and child.

## Results

**Figure 1:**
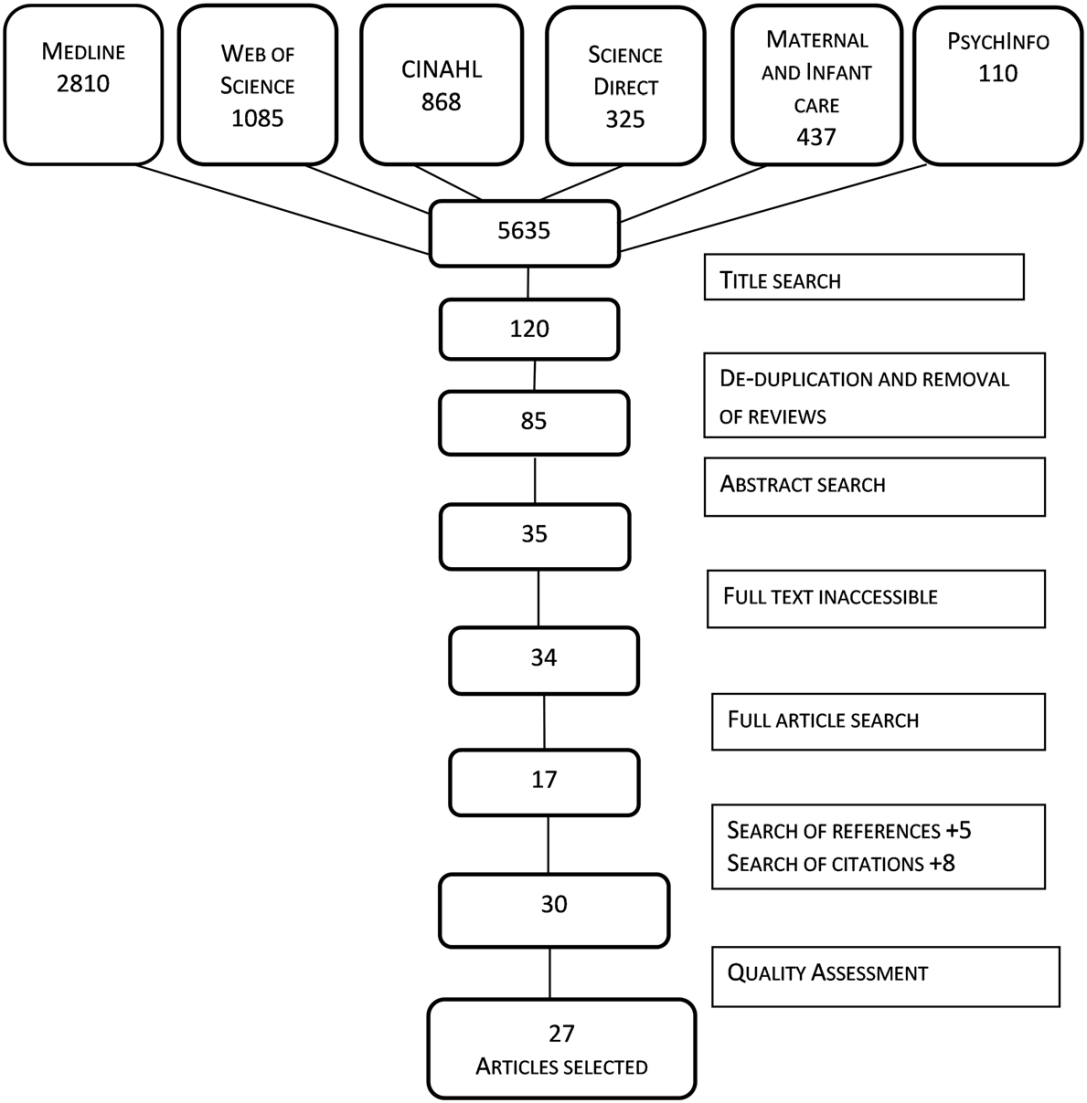
Systematic review selection process.

The study selection process is outlined in Fig. 1; 5635 articles were returned. Their titles were screened by the first author according to the inclusion and exclusion criteria (Table 2), and irrelevant papers were discarded (*n* = 5515). Duplicate and review articles were then removed (*n* = 35). The abstracts of the remaining articles (*n* = 85) were screened for relevance, and exclusion and inclusion criteria were applied resulting in 50 articles being discarded.

**Table 2:** Inclusion and exclusion criteria

Inclusion criteria	Exclusion criteria
– Qualitative or quantitative	– Studies with a non‐human population
– Peer reviewed	– Studies with a primary focus on children over the age of 2 years
– Studies of feeding behaviours in typically developing children aged 0–2 years	– Studies with a primary focus on maternal (rather than infant) feeding behaviours
– Studies of hunger and satiation cues in typically developing children in the first 2 years of life	– Studies with a primary focus on feeding in premature infants
	– Studies relating to infant or toddler feeding in populations with specific medical conditions (e.g. cystic fibrosis, developmental disorder, exposure to maternal substance abuse and cleft lip)
	– Studies with a primary focus on infant or toddler feeding in populations with maternal disorder (e.g. depressive illness and eating disorder)
	– Review articles/books
	– Papers not written in English
	– Papers where the full text version is unavailable

Of the remaining 35 articles, only 34 were fully accessible. These were read in full, and those not fulfilling inclusion and exclusion criteria were discarded (*n* = 17). A reference list search and a citation search were conducted for the remaining 17 articles. From these, a further 13 articles were obtained that met the inclusion/exclusion criteria (Table 3). The remaining 30 articles were subjected to quality assessment. Those scoring below 11 on the 22 point scale were removed, and a final 27 articles were selected.

**Table 3:** Selected papers

Authors and title	Participants and sample	Design and methods	Main findings	Implications for understanding hunger and satiation in infancy	Quality ratings first and*second* raters
Anderson et al. (2001)	*N* = 29	**Cross‐sectional**	Introduction of solids was based on infant age, size, weight and a variety of increased infant hunger cues.	Both infant behaviours (chewing hands and crying) and infant characteristics (age and size) are used by mothers to determine feeding state along with external cues such as time.	**20/22**
‘Rattling the plate – reasons and rationales for early weaning’	Multiparous and primiparous mothers, mean age 27 years, of babies aged between 8 and 18 weeks, mean age 13 weeks.	Focus group discussions exploring beliefs and attitudes regarding the introduction of solid food. Qualitative content analysis.			***19/22***
Blossfield et al. (2007)	*N* = 70	**Quasi‐experimental**	Variability in consumption of chopped carrots related to familiarity with different textures, higher dietary variety, food fussiness and the number of teeth infants possessed. Amount eaten was associated with level of enjoyment.	Amount of food consumed varies according to liking as well as with infant characteristics (e.g. pickiness or number of teeth).	**22/22**
‘Texture preferences of 12‐month‐old infants and the role of early experiences’	39 male and 31 infants aged between 48 and 57 weeks, mean age 52.7 weeks.	Infants fed chopped or pureed carrots. Measures – amount of food consumed, maternal ratings of enjoyment and questionnaire measures, e.g. CEBQ* and FFQ.†			***22/22***
Darlington & Wright (2006)	*N* = 75	**Short‐term longitudinal**	Slow weight gain was significantly associated with fearful temperament. Fast weight gain was associated with irritable behaviour.	Infant temperament may affect appetite or the communication of hunger, although mothers may feed irritable babies more in order to soothe them.	**20/22**
‘The influence of temperament on weight gain in early infancy’	43 male and 32 female infants between 8 and 12 weeks of age, mean age 10 weeks.	Infants' birthweights and weights taken at 8–12 weeks. Completion of IBQ‡ and Baby's Day record by mothers.			***20/22***
Forestell & Mennella (2012)	*N* = 92	**Experimental**	Infants with high scores on the approach dimension of temperament ate more of a test vegetable for longer and with fewer negative expressions.	Infant temperament may play a part in food acceptance and amount consumed. Consumption is therefore not purely determined by hunger.	**22/22**
‘More than just a pretty face. The relationship between infant's temperament, food acceptance, and mothers' perceptions of their enjoyment of food’	48 male and 44 female infants, mean age 52 weeks.	Infants video‐recorded when fed test vegetable in laboratory conditions. Measures: facial expression coding; Infant Temperament Scale and maternal ratings of infants' enjoyment.			***21/22***
Gross et al. (2010)	*N* = 368	**Cross‐sectional**	Hand sucking was viewed as a hunger cue and head turning as a satiation cue. Most mothers (72%) believed crying must be an indication of hunger. Most (93%) also believed their babies could sense their own satiety. Mothers with high BMIs and low educational levels appeared less sensitive to satiation cues.	Common cues are used by mothers to identify hunger and satiation. Lower maternal educational level and higher BMI may be associated with lower awareness of infant satiation.	**19/22**
‘Maternal perceptions of infant hunger, satiety, and pressuring feeding styles in an urban Latina WIC population’	Mothers, mean age 28 years with infants aged < 20 weeks, mean infant age 18.8 weeks.	Secondary analysis of survey data regarding maternal perceptions of hunger, satiation and pressuring feeding style.			***17/22***
Hodges et al. (2008)	*N* = 71	**Cross‐sectional**	Mothers' responsiveness to feeding cues was variable. Some focused on amount consumed, while others focused on infant state or oral behaviours. Specificity of cues increased with infant age.	A range of overt and subtle hunger and satiation cues are reported by mothers, e.g. crying, licking the lips, spitting food out and stopping the meal. Different mothers focused on different cues.	**19/22**
‘Maternal decisions about the initiation and termination of infant feeding’	Mothers of full term infants at 12, 26 or 52 weeks of age, 35 males and 36 females. Mean maternal age, 28.9 years.	Structured interviewing and qualitative content analysis.			***17/22***
Hodges et al. (2013)	*N* = 144	**Cross‐sectional**	Mothers responded more to hunger than fullness cues. Responsiveness to cues was associated with maternal characteristics (education, BMI and breastfeeding duration). Mothers were more responsive to hunger cues in older self‐feeding children.	Hunger cues may be more salient for mothers than satiation cues. Mothers appear more responsive to the cues of older children. Responsiveness to satiation appears to be associated with higher educational level, lower BMI and longer breastfeeding duration.	**22/22**
‘Development of the responsiveness to child feeding cues scale’	Mothers of 28 to 104‐week‐old infants and toddlers, mean maternal and infant age and M : F ratio unknown.	Development and testing of an observational measure of caregiver responsiveness to child feeding cues using structured observation of infant/toddler feeding.			***21/22***
Hwang (1978)	*N* = 58	**Short‐term longitudinal**	On day four, mean number of feeding periods was significantly higher for male than female infants. The first feeding period on day four was significantly longer for females than males. During feeding on both days, male infants cried more than females.	Newborn male and female infants appear to show different feeding behaviours, with possible implications for maternal perceptions of hunger and satiation.	**14/22**
‘Mother–infant interaction – effects of sex on infant feeding behavior’	Primiparous mothers 23 male and 35 female newborn infants observed at <1 week (2 and 4 days). Maternal age unknown.	Time sampled observation of two single breastfeeding sessions on days two and four of life in hospital setting.			***15/22***
Lew & Butterworth (1995)	*N* = 18	**Cross‐sectional**	No difference found between the distribution of hand–face and hand–mouth contacts pre‐feed. Proportion of hand–mouth contacts was not greater before feeding than after feeding. Open mouth postures before hand–mouth contacts only occurred before feeding.	Open mouth postures prior to hand mouth contacts may be an indication of hunger in newborn infants.	**21/22**
‘The effects of hunger on hand–mouth coordination in newborn infants’	Newborn term infants born between 38 and 42 weeks gestational age observed at 1 week or younger.	Structured observations of infants before and after milk feeding by formula or breast. Analysis of differences between hand–face and hand–mouth contacts.			***20/22***
Llewellyn et al. (2011)	*N* = 2402	**Cohort study**	Four appetite constructs were identified – food responsiveness, enjoyment of food, satiety responsiveness and slowness in eating. All constructs had good internal reliability and correlated with ‘general’ appetite. Group differences were observed in relation to appetitive behaviours.	Different groups of infants have different appetitive behaviours; e.g., males appear to have larger appetites and to be less satiety responsive than females; premature infants have smaller appetites and higher satiety sensitivity than term infants; and breastfed infants appear less satiety responsive than formula‐fed infants.	**21/22**
‘Development and factor structure of the Baby Eating Behaviour Questionnaire in the Gemini birth cohort’	1194 male and 1208 female infants, mean age 32.8 weeks, range 16–80 weeks.	BEBQ§ for milk‐fed infants. Questionnaire items refined via interviews with a sample of mothers (*n* = 10).			***21/22***
Llewellyn et al. (2012)	*N* = 4634	**Cohort study**	Infant weight was correlated with BEBQ appetite traits. Genetic influence was shown for satiety responsiveness, slowness in eating and appetite.	Eating traits of infants are heritable. Expression of appetite is therefore influenced by genotype.	**21/22**
‘Inherited behavioral susceptibility to adiposity in infancy: a multivariate genetic analysis of appetite and weight in the Gemini birth cohort’	2289 males and 2345 female infants, mean age 32.8 weeks, range 16–80 weeks.	BEBQ§ and infant weight measures taken at 12 weeks + multivariate genetic modelling.			***21/22***
McMeekin et al. (2013)	*N* = 698	**Cross‐sectional**	Mothers of infants with difficult temperaments reported a lower awareness of hunger and satiation cues and were more likely to use food to soothe.	It may be difficult for mothers of infants with difficult temperaments to distinguish hunger and satiation cues from other kinds of distress. Maternal depression also appears to be associated with lower awareness of infant feeding cues and greater use of food to calm babies.	**21/22**
‘Associations between infant temperament and early feeding practices. A cross‐sectional study of Australian mother–infant dyads from the nourish randomised controlled trial’	342 male and 356 female infants between 8 and 28 weeks of primiparous mothers. Mean infant age 17.2 weeks. Mean maternal age 30.1 years.	Maternal self‐report on STSI¶ and IFQ.∥			***20/22***
Mennella et al. (2001)	*N* = 46	**Experimental**	Infants exposed to carrot flavours *in utero* or during lactation exhibited fewer negative facial expressions to carrot‐flavoured cereal than plain cereal. Infants exposed to carrot flavour *in utero* were perceived by mothers to enjoy carrot‐flavoured cereal more than plain cereal.	Previous exposure to flavour leads to greater acceptance, greater enjoyment and greater consumption. Amount eaten is not purely determined by hunger. Facial expression may be one way of differentiating between cessation of eating due to dislike and that arising from satiation.	**21/22**
‘Prenatal and postnatal flavor learning by human infants’	28 male and 18 female infants. Mean infant age 22.6 weeks.	Infants assigned to one of three groups involving drinking carrot juice or water during pregnancy and breastfeeding. Responses to cereals containing water or carrot juice were measured via facial expression coding, maternal ratings of enjoyment and amount consumed.			***20/22***
Mennella et al. (2009)	*N* = 97	**Experimental**	Type of formula fed to infants impacted on responses to different tasting cereals. Formula‐fed infants eating complementary foods showed preferences for the tastes of foods to which they had already been exposed.	Prior exposure leads to greater consumption of food with familiar taste compounds. Negative facial expression may provide a basis for distinguishing between satiation and dislike.	**21/22**
‘Early milk feeding influences taste acceptance and liking during infancy’	Full term infants between 16 and 36 weeks, mean age 25 weeks, who had been spoon fed baby cereal for at least 2 weeks.	Subgroups of breastfed and two types of formula‐fed babies were observed on different occasions to measure acceptance of sweet, salty, bitter, savoury, sour and plain cereals.			***21/22***
Nisbett & Gurwitz (1970)	(Experiment 1)	**Experimental**	Heavy infants were more responsive than medium and light weight infants to sweetened formula. Female infants responded more to sweetened formula than males. Heavier and female infants consumed significantly less in the small hole condition. Medium weight, lighter weight and male infants' consumption was not significantly affected by this condition.	Sex and weight may impact on satiety responsiveness to sweetened milk. Sex and weight may impact on effort expended in feeding and consequent amount consumed.	**18/22**
‘Weight, sex, and the eating behavior of human newborns’	*N* = 42	Infants in three weight groups were alternately fed a sweet and standard formula of the same energy density daily at the same time. Intake per feed was recorded. Infants were formula fed over 2 days with a normal or small hole teat. Mothers recorded consumption and time at which feeds began and ended.			***17/22***
	22 male and 20 female newborn infants.				
	(Experiment 2) *N* = 34				
	18 male and 16 female newborn infants.				
Parkinson & Drewett (2001)	*N* = 100	**Cross‐sectional**	Despite similarity in the age of the toddlers self‐feeding and being fed varied highly. Intake was correlated with number of bites rather than meal duration. Self‐feeding led to a longer meal time on average, while longer meals were associated with lower food intake.	Number of bites may be a better indication of hunger levels than meal duration, although account needs to be taken of whether the child self feeds or is fed by the mother. Self‐feeding tends to lead to longer meal duration and lower intake in toddlers.	**20/22**
‘Feeding behaviour in the weaning period’	Mother infant dyads. 51 male and 49 female. Infants/toddlers observed between 52 and 61 weeks, mean age 55 weeks. Maternal age range ≤24 to ≥35 years.	Naturalistic observation of two meal times analysed using all occurrence sampling. Codes developed regarding mothers' feeding of children and child self‐feeding and related child behaviours.			***17/22***
Paul et al. (1996)	*N* = 20	**Short‐term longitudinal**	Two‐week‐old infants were visually attentive when feeding. Motor activity and alertness shifted from pre‐feeding to post‐feeding time during the first 6 months.	Motor behaviours differ with feeding state and at different points in the feeding cycle according to infant age. Differences also appear to exist in the sucking behaviours and consumption patterns of formula‐fed and milk‐fed babies.	**15/22**
‘Infant feeding behavior: development in patterns and motivation’	Full term infants at 2, 10, 18 and 26 weeks. Sex unknown.	Structured observation prior to during and after milk feeding. Observations supported by video and polygraphic recording of behaviours such as sucking, breathing and swallowing.			***13/22***
Reau et al. (1996)	*N* = 281	**Cross‐sectional**	No differences were reported between feeding time in terms of birthweight or birth order. 90% of infants and toddlers took fewer than 30 min to eat a meal. Reports of feeding problems were especially common in toddlers.	Feeding problems are common in infants and especially toddlers. Variability in hunger is normal. Meal durations beyond 30 min may indicate feeding problems.	**17/22**
‘Infant and toddler feeding patterns and problems: normative data and a new direction’	157 male and 124 female infants and toddlers, age range 12–108 weeks. Mean age unknown.	Survey research using an unvalidated self‐report questionnaire. Questionnaire items included infant and toddler hunger at the start of a meal, feeding behaviours, feeding problems and feeding duration.			***14/22***
Skinner et al. (1998)	*N* = 98	**Longitudinal**	Hunger communication appeared before satiation (4.4–5.7 and 5.8–7.5 months, respectively). Extreme variability was identified in communicative behaviours at meal times. Food likes and dislikes increased with age as did verbal communication relating to eating.	Hunger and satiation communication is highly variable. Likes/dislikes are easier to discern in older infants than younger ones, although liking was exhibited less than dislike through facial expression.	**19/22**
‘Mealtime communication patterns of infants from 2 to 24 months of age’	Infant mother dyads. Typically developing infants from 8 to 96 weeks. Infant sex, mean infant age and mean maternal age unknown.	Structured interviews and researcher administered questionnaire at 10 time points from 2 to 24 months. Participants were randomly assigned to six interviews. Data were collected regarding infant and toddler mealtime communication at each time point.			***15/22***
Stevenson et al. (1990)	*N* = 34	**Cross‐sectional**	Feeding outcomes were similar for both groups. Pre‐term infants fussed more during feeding than term infants. Vocalisations did not differ between groups. However, mothers of premature babies responded to vocalisations with offers of food more than mothers of term infants.	Expression of hunger may differ subtly in premature babies. Mothers of these babies offer food more in response to vocalisation than those of full term babies.	**17/22**
‘Rhythms in the dialogue of infant feeding: preterm and term infants’	Mother infant dyads with 17 pre‐term infants and 17 full term infants at 32 weeks (age gestationally adjusted). Maternal age and infant sex unknown.	Solid food feeding interactions video‐recorded through a one‐way mirror. Coding of maternal and infant behaviours such as gaze, vocalisation and self‐feed.			***15/22***
Turkewitz et al. (1966)	*N* = 35	**Cross‐sectional**	The proportion of hand flexion to extension movements was greater prior to feeding than post‐feeding, regardless of whether infants were awake or asleep.	Hand flexion appears to be associated with hunger in new born infants.	**17/22**
‘Relationship between feeding condition and organization of flexor–extensor movements in the human neonate’	Newborn female infants, aged < 1 to 1.5 weeks, mean age ≏ 1 week	Observational study of flexion and extension movements of infants' hands during two 5‐min periods prior to and post‐feeding.			***19/22***
van Dijk et al. (2009)	*N* = 20	**Short‐term longitudinal**	Amount consumed per meal increased over time. Mealtime duration was stable across time (average 8 to 10 min). Frequency of food refusals decreased over time. Variability was found in feeding behaviours both across and within infants particularly during the period after the introduction of solids.	Infant feeding behaviour is highly variable during the weaning period; however, meal duration increases over time. Food refusal is also common during weaning.	**19/22**
‘Variability in eating behavior throughout the weaning period’	12 male and 8 female full term infants aged between 16 and 24 weeks, mean age 22 weeks.	Naturalistic observation of infants and caregivers across a 12‐week period following the introduction of solids. Feeding video‐recorded and coded.			***17/22***
Ventura et al. (2012)	*N* = 30	**Experimental**	Infants consumed significantly less cows' milk formula and showed higher satiety ratios after the enhanced cows' milk formula and the protein hydrolysate than standard cows' milk formula.	Formula composition impacts on both satiation and satiety regardless of energy content. The study potentially offers five means of identifying hunger and satiety in a research context: amount of milk initially consumed, rate of consumption, response to additional offers of milk, compensation feeding at subsequent meal and satiety ratio.	**21/22**
‘Infant regulation of intake: the effect of free glutamate content in infant formulas’	14 male and 16 female infants, mean age 8.5 weeks.	Infants were fed one of three different formulas over 3 days: cows' milk formula, a protein hydrolysate formula and cows' milk formula with added free glutamate. Satiety ratios were calculated for each formula.			***20/22***
Wasser et al. (2011)	*N* = 217	**Cross‐sectional**	Infants with high distress to limitations were more likely to receive solid foods early. Maternal obesity was associated with early introduction of solids, and maternal depression was associated with the early introduction of juice.	Infants with difficult temperaments may be perceived to be hungrier or may be fed to soothe them. Infants with difficult temperaments may also be given juice to soothe them or as a coping response by depressed mothers. Obese mothers may misinterpret difficult temperament for hunger or may have larger, hungrier babies.	**22/22**
‘Infants perceived as “fussy” are more likely to receive complementary foods before 4 months’	Infant mother dyads visited at 12, 24, 36, 48 and 72 weeks of infant age. 101 males and 116 females. Mean maternal age 22.7 years.	Infant feeding patterns assessed thorough dietary history and 24‐h dietary recall. Infant temperament traits measured by Infant Behaviour Questionnaire – revised			***22/22***
Wright et al. (1980)	*N* = 190	**Short‐term longitudinal**	Where long intervals occurred between feeds, breastfed infants consumed a larger meal than formula‐fed infants. Differences were noted in the sucking pattern of breastfed and formula‐fed infants. Over the first 2 months, diurnal differences appeared in the size of feed consumed in breastfed but not formula‐fed infants.	Breastfed and formula‐fed babies show different patterns of feeding behaviour in terms of sucking behaviour and variability of consumption.	**15/22**
‘Do breast‐feeding mothers know how hungry their babies are?’	132 formula‐fed and 58 breastfed infants in the first 8 weeks of life. Infant sex unknown.	Video recording of feeding sessions at monthly intervals from just after birth for formula and breastfed infants. Mothers also kept diaries of infants' feeds.			***15/22***
Wright (1986)	*N* = 30	**Short‐term longitudinal**	77% of mothers reported their infants' hunger varied across the day, more so for boys than girls. Milk intake did not vary significantly across the day, and no significant difference was reported between boys' and girls' milk consumption. Mothers' ratings of hunger correlated with those for intake for 9 of the 14 mothers.	Most breastfeeding mothers were able to assess accurately their infant's hunger. However, infant sex may exert an influence on mothers' interpretation of hunger cues. Mothers of boys may misinterpret high activity and arousal levels as hunger.	**15/22**
‘The development of differences in the feeding behaviour of formula and breastfed human infants from birth to 2 months’	Mothers of 14 male and 16 female breastfed infants, mean infant age 4 weeks. Mean maternal age unknown.	Mothers asked three questions regarding infant hunger. 14 mothers also kept a 4‐day diary of feeds, provided hunger ratings and weighed infants before and after feeds.			***16/22***
Young & Drewett (2000)	*N* = 30	**Short‐term longitudinal**	Median meal duration was 17 min. There was high variability between infants in feeding behaviours and across meals. Refusal was a common but highly variable behaviour – median 11, range 0–101.	At 52 weeks of age, toddlers' eating behaviour is variable across meals. Food refusal is common in this age group. Toddlers also consume desserts faster and with fewer refusals than main courses.	**19/22**
‘Eating behaviour and its variability in 1‐year‐old children’	13 female and 17 male infants aged 50–57 weeks old. Mean age unknown.	Video‐recorded observations of meals in the home over 2 consecutive days coded with a scheme developed from two other studies.			***18/22***

* Child Eating Behaviour Questionnaire.

† Food Frequency Questionnaire.

‡ Infant Behaviour Questionnaire.

§ Baby Eating Behaviour Questionnaire.

¶ Short Temperament Scale for Infants.

∥ Infant Feeding Questionnaire.

### Quality assessment of studies

In the final stage of selection, articles were rated for quality using a tool developed by Moore (2012). The tool was selected on its suitability for assessing both qualitative and quantitative papers and non‐intervention studies. Quality ratings were subjected to inter‐rater reliability analysis using a non‐fully crossed design; the main author rated all papers, while second authors each rated a different subset of papers. A random sample of 14 papers was selected for the intraclass correlation analysis. A high level of inter‐rater agreement was found (single measures intraclass correlations by use of a one‐way random effects model), *r* = 0.82 (*P* < 0.001).

### Overview of selected papers

#### Terminology

Several selected studies use the terms satiety and satiation synonymously (e.g. Hodges et al. 2008; Llewellyn et al. 2012). This review distinguishes between these with ‘satiation’ referring to the process leading to the cessation of eating and ‘satiety’ referring to the feeling of fullness after eating that determines the interval before the next meal (Blundell & Bellisle, 2013).

#### Summary of selected studies

The main methodological features of the selected studies are reported in Table 3. Most studies were cross‐sectional (*n* = 11); others had longitudinal/repeated measures components (*n* = 8) or were experimental/quasi‐experimental (*n* = 6). Two studies were cohort studies. These involved questionnaires and one used modelling of heritability of eating traits (Llewellyn et al. 2012). Most of the cross‐sectional studies employed surveys and structured observational methods. The exceptions to this were Hodges et al. (2008) and Anderson et al. (2001) who used semi‐structured interviews and focus groups, respectively.

#### Areas of investigation

Six main research areas were identified in the retrieved studies, as indicated below. A summary of findings from the selected papers appears in Table 3.

1. Maternal perceptions of infants' and toddlers' hunger and satiation communications.
2. Movement and sucking behaviours associated with hunger and satiation.
3. Impact of infant characteristics on the expression and perception of hunger and satiation.
4. Feeding behaviour norms in infancy.
5. Feeding method, composition of milk, hunger, satiation and satiety.
6. Infant food preferences, how these are expressed and implications for understanding hunger and satiation.

### Findings

#### Maternal reports of feeding cues

Several studies have investigated mothers' perceptions of infants' feeding cues. Anderson et al. (2001) used focus groups to examine maternal beliefs regarding readiness for weaning. In this context, perceptions of hunger related both to babies' characteristics (e.g. age and weight) and their behaviour (e.g. increased rate of milk consumption, agitation and changed sleeping patterns). Mothers also reported being able to identify a ‘hungry cry’; however, this was differentiated from other cries by time of day rather than the characteristics of the cry itself. Reported satiation cues included the baby seeming more ‘content’ and them wishing to eat less often.

Gross et al. (2010) also examined mothers' perceptions of infant hunger and satiation. In a survey relating to general feeding rather than weaning, they found mothers were attentive to four hunger and satiation behaviours: hand sucking, head turning, crying and babies ‘knowing’ they were full. The list of cues was generated by the authors, although participants agreed they used them to identify hunger and satiation. Gross et al. (2010) also found associations between certain maternal characteristics and perceptions of feeding cues: obese mothers were less likely to agree that babies could sense their own satiation, and maternal obesity and longer breastfeeding history were associated with perceiving hand sucking as indicating hunger.

In a study involving semi‐structured interviews, Hodges et al. (2008) investigated cues that prompted mothers to initiate and end feeding. Like Anderson et al. (2001), the authors found mothers used both infant behaviours and external cues (e.g. time) to identify hunger. Commonly identified hunger cues in this study were crying, fussing and licking the lips, and these were reported across several age groups (3, 6 and 12 months). Commonly reported satiation cues included pulling away, spitting food out and stopping feeding. The authors also found that the prominence, intensity and specificity of infant cues guided decisions about initiating and ending feeds and that mothers found cues easier to interpret with increasing infant age.

In a later study, Hodges et al. (2013) described the development of the Responsiveness to Child Feeding Cues Scale (RCFCS). In devising this, the authors identified 20 types of hunger cue and 28 types of satiation cue. Hunger and satiation cues were further categorised as ‘early’ (e.g. increased alertness), ‘active’ (e.g. excitatory movements) and ‘late’ (e.g. fussing and crying) in order to reflect changes in cue intensity. Satiation cues were not described directly in the study, although the authors found mothers' responsiveness to satiation to be predicted by certain maternal characteristics (lower BMI, longer breastfeeding duration and higher educational level). They also found mothers to be more responsive to hunger than satiation cues.

The only longitudinal study retrieved in the search was conducted by Skinner et al. (1998). They examined mealtime communication behaviours in infants and toddlers using structured interviews with mothers. The authors found that hunger behaviours, e.g. opening the mouth for the spoon, appeared at a younger age than satiation behaviours, e.g. closing the mouth to reject food (4.4 to 5.7 months vs. 5.8 to 7.5 months, respectively). They also noted that overall hunger and satiation behaviours were highly variable across infants. The study also examined infants' communication of food likes and dislikes. Findings relating to this are discussed alongside research relating to food preferences.

Wright (1986) also observed variability in the expression of hunger by infants although this time by infant sex. Mothers of breastfed babies were asked when their infants were most hungry, how they identified hunger and also about the variability of their breastmilk supply. All mothers of male babies agreed hunger varied across the day, but only around half the mothers of females reported this. Mothers identified increased frequency of feeding as a hunger cue for males, whereas agitation was cited for females. Late afternoon and early evening were identified as hungry times for males, while mothers of females associated hungry times with feeling they had less breast milk, rather than time of day. Despite such differences, recordings of infant weight taken from before and after feeding indicated that relatively constant volumes of milk were consumed by girls and boys across the day. It appears then that mothers of male and female infants may interpret different behaviours as hunger depending on the sex of their child (Wright 1986).

#### Movement and sucking behaviours associated with hunger and satiation

A few studies have involved observations of infants under controlled conditions before, during and after feeding. Lew & Butterworth (1995) observed hand to mouth contacts in newborns pre‐prandially and post‐prandially. They found that hunger did not affect where hand contacts were made on the face, and there was no difference between the proportion of hand–mouth contacts before and after feeding. However, hand–mouth contacts preceded by open mouth postures were only observed before feeding. This coordination of open mouth postures with hand–mouth contacts may therefore be associated with hunger in newborns.

Similarly, Turkewitz et al. (1966) examined hand movements before and after feeding. The researchers observed the flexion and extension movements of newborns' hands and found that regardless of whether infants were awake or asleep, the proportion of flexion movements was significantly greater before feeding than after. Flexed hand postures may therefore be another behavioural indication of hunger in young infants.

While Turkewitz et al. (1966) and Lew and Butterworth (1995) investigated infant hand movements before and after feeding, Paul et al. (1996) examined several aspects of pre‐prandial and post‐prandial behaviour. They video‐recorded milk feeds in infants at 8‐week intervals in infants between 2 and 26 weeks of age. The researchers found sucking behaviours increased in rate with infant age, while the number and length of pauses in sucking decreased. Breast and formula feeding behaviours were compared at 2 weeks of age but not beyond this; breastfed infants consumed milk at less than a third of the rate of formula‐fed babies, and breast feeds took around four times longer than formula feeds. The authors also examined motor activity during feeding. This was low for all age groups (Paul et al., 1996). Following feeding, motor activity and muscle tone decreased in 2‐week‐old infants. However, post‐feeding motor activity increased in older infants. The study therefore indicates that infant sucking and motor activity vary with hunger and satiation, although the precise pattern of behaviour differs with infant age and feeding method.

#### Effect of infant characteristics on hunger, satiation and feeding behaviour

Several studies have examined associations between infant characteristics and feeding behaviour. Using the Infant Temperament Scale (Carey & McDevitt, 1978), Forestell and Mennella (2012) investigated associations between temperament and liking of a novel vegetable. They found that infants with higher ratings on ‘approach’ traits (those more willing to approach novel situations) ate more green beans, and for longer, and showed fewer negative facial expressions (assessed by mothers) than those with lower approach ratings.

Darlington & Wright (2006) also investigated the impact of temperament on feeding although in relation to weight gain in the first 2 months of life. Using the Infant Behaviour Questionnaire (IBQ) (Rothbart, 1981), they found that infants with high fearfulness scores exhibited slow weight gain, while those with high scores on ‘distress to limitations’ showed faster weight gain. The IBQ was also used to investigate infant temperament and the early introduction of complementary feeds by low‐income mothers by Wasser et al. (2011). They noted that both ‘distress to limitations’ and infant ‘activity level’ were significantly associated with the introduction of solids before 4 months of age. In addition, Wasser et al. found maternal obesity to be significantly associated with the early introduction of solids, suggesting again that maternal characteristics may influence perceptions of infant hunger.

Research by McMeekin et al. (2013) further supports the contention that both infant and maternal characteristics influence perceptions of feeding cues. In a study using the Short Temperament Scale (STSI) (Sanson et al. 1987), they found that mothers of babies with ‘difficult temperaments’ were significantly more likely to feed their babies to calm them. Meanwhile, regarding maternal characteristics, mothers with higher scores on the Edinburgh Post Natal Depression Scale (Cox et al. 1987) were found to be significantly less aware of infant feeding cues and more likely to feed their babies to calm them.

Llewellyn et al. (2011) also explored the impact of infant characteristics on feeding behaviour. In developing the Baby Eating Questionnaire (BEBQ), they examined associations between individual characteristics and feeding traits. Male babies were found to have larger appetites, to respond more to food cues and to be less satiety responsive (sensitive to feeling full and fullness between meals) than females. Premature infants were reported to have smaller appetites, lower enjoyment of food, slower feeding and higher satiety responsiveness than full term infants. Breastfed babies had larger overall appetites, were more responsive to food cues and were less sensitive to satiety cues than mixed‐fed or formula‐fed babies. Finally, infants with higher birthweights had larger appetites, fed more quickly, enjoyed food more and were less responsive to satiety than lower birthweight babies. Thus, sex, birthweight and gestational age at birth may all influence infant appetite and feeding cues.

The BEBQ was also used by Llewellyn et al. (2012) to investigate relationships between genotype and eating traits. In this large scale twin study, details of zygosity, infant age, gestational age and sex were collected alongside appetite data at the age of 3 months. Significant shared genetic effects were found in twins regarding weight, slowness in eating traits, satiety responsiveness and appetite size. The findings therefore suggest that appetite and behaviours associated with this are shaped in part by individual genetic make‐up.

Additional evidence that gestational age at birth influences feeding behaviour comes from research by Stevenson et al. (1990). They observed feeding behaviour in term and pre‐term infants. No significant differences were found between groups regarding amount eaten, infant vocalisations or infants' gaze at mothers during feeding. However, pre‐term infants were significantly fussier during feeding than term infants, and mothers of pre‐term infants responded to vocalisations with offers of food, while mothers of term infants did not.

In relation to sex and feeding cues, an observational study of newborns by Hwang (1978) found that on the fourth day of life, boys suckled significantly more frequently and for shorter periods than girls. In addition, Hwang noted that, during feeding, males were significantly more likely to fuss than females, both on the second and fourth days after birth. Nisbett & Gurwitz (1970) also reported sex differences in feeding behaviour although within the context of formula feeding. They increased the sweetness of formula fed to newborns and found female and heavier infants consumed significantly more sweetened formula than male or lighter infants. In a second experiment, the researchers manipulated the size of the hole in the bottle teat, alternating feeds of standard formula with a regular and a small hole. Consumption by boys was not affected by the small hole, although that of female and heavier babies was reduced. The findings suggest that female and heavier infants may be more responsive to sweetness or, possibly, are more able to detect this. Female and heavier infants may also be less willing to expend energy on feeding when this is made more difficult.

#### Infant feeding behaviour norms

A number of studies have examined normative aspects of infant feeding such as intake and duration of feeding. These provide contextual information that is helpful in understanding feeding behaviour and the expression of feeding cues in infancy. In an observational study of toddlers, Parkinson & Drewett (2001) found that mean meal duration across two observed meals was approximately 19 min with a mean intake of 165 g. However, within these parameters, the authors found a high degree of variability across individuals and meals. They found meal duration and intake were not significantly correlated, but instead, intake increased significantly with number of bites. Number of bites therefore may be a better indicator of level of hunger in toddlers than meal duration.

Infant and toddler feeding norms were also investigated by Reau et al. (1996). They asked mothers about duration and enjoyment of eating, food refusal and eating speed. Mean reported feeding duration did not differ significantly across age, birthweight or birth order; 90% of infants and toddlers were reported to finish a meal in less than 30 min. Food refusal, however, was commonly reported in toddlers, indicating that this is not necessarily a satiation cue but rather a developmentally typical eating behaviour in toddlers.

Young & Drewett (2000) conducted observational research into toddlers' eating behaviour. Their work provides particular insights into feeding behaviour in the contexts of savoury and sweet courses. Median intake for desserts and mains was similar (71 and 82 g, respectively), although median durations were 5 and 10 min, respectively. Furthermore, median number of food refusals for sweet courses was around half that for the main course, indicating that the children consumed desserts more quickly and with fewer refusals than in main courses despite already being partly satiated.

Other observational research into infant eating patterns was carried out by Van Dijk et al. (2009), in this case in the specific context of weaning. They found considerable variability within individuals in terms of food refusal, intake and meal duration. As might be expected, this variability was greatest in the earliest spoon feeding sessions. The average duration of meals was relatively constant (8 to 10 min across the 3‐month period observed). Consumption, however, increased during the first 12 weeks of weaning, while refusal decreased. This study provides further evidence then that developmental stage impacts on behaviours associated with hunger and satiation.

#### The impact of milk composition and feeding method on infant feeding behaviour

While Paul et al. (1996) and Llewellyn et al. (2011) reported incidental differences in feeding behaviour according to feeding method, two studies have examined relationships between feeding method and feeding behaviour more directly.

It has been proposed that differences between breastfed and formula‐fed infants in growth velocity and in the experience of hunger and satiation may be attributable in part to milk composition. Breast milk differs from formula in amount and form of amino acid content, and this may play a role in the faster weight gain recorded in infants fed cow's milk formula compared with breast milk. Because free amino acids such as glutamate are implicated in satiation in both animal and human studies, Ventura et al. (2012) manipulated milk composition to examine its effects on intake and satiety. They fed infants a standard cows' milk formula, a high free glutamate formula or a cow's milk formula fortified with free glutamate. Infants consumed significantly less of the high free glutamate formula and the fortified cows' milk formula than the regular cow's milk formula. The authors also examined the effect of formula composition on satiety (determined by the effect of the first meal on later consumption). They found significantly higher levels of satiety after consumption of the high free glutamate formula and the fortified cows' milk formula than standard cow's milk formula.

The impact of milk composition on feeding behaviour is also evident in research by Wright et al. (1980) in relation to breast milk and formula milk. These authors video‐recorded three feeding sessions at monthly intervals from birth to 2 months in both formula and breastfed infants. Mothers also kept feeding diaries. Breastfed babies exhibited pauses in sucking while feeding, whereas formula‐fed infants fed almost continuously. The authors also identified diurnal variations in the size of feed consumed by breastfed infants, with early morning feeds being the largest of the day. This may represent a diurnal variation in breastmilk composition or in the hunger or thirst of breastfed babies; however, it was not observed in formula‐fed babies. Feeding method (breastfeeding or formula) therefore appears to impact both on feeding behaviours and patterns of hunger.

#### Food preferences and infant feeding behaviour

Several studies suggest that hunger and satiation are not the only drivers for infant consumption but that hedonic responses to food also play a role. Mennella et al. (2009) investigated acceptance of cereal flavoured with breast milk, cow's milk formula and hydrolysed casein formulas (HCFs) in 4‐ to 9‐month‐old infants. HCFs have stronger savoury, bitter and sour tastes than breastmilk or cow's milk formula, and the investigators found that infants previously fed on these ate significantly more savoury, sour and bitter tasting cereals than those breastfed or fed cow's milk formula. Mennella et al. (2009) also assessed liking of the cereals via the Facial Action Coding System (Ekman & Friesen, 2002). Infants in this study showed fewer negative facial expressions (e.g. brow lowering, nose wrinkling and squinting) than the other groups. Thus, enjoyment of taste (shown through facial expression) was significantly associated with amount consumed.

Food preference research has examined flavour as well as taste preference. Mennella et al. (2001) examined liking of carrot flavour in breastfed infants of mothers who drank carrot juice or water during pregnancy and lactation. Infants with previous exposure to carrot flavour *in utero* or through breastfeeding showed fewer negative facial expressions and greater enjoyment of carrot juice‐flavoured cereal (rated by mothers) than those without prior exposure. Amount consumed and meal duration showed a similar trend, but these were not significant. In terms of food flavour (rather than taste), infants may communicate liking or dislike through facial expression more than intake or eating duration.

While several studies have examined taste and flavour preferences in infancy, the impact of texture preference on eating has been reported by Blossfield et al. (2007). They used mothers' ratings to assess enjoyment of chopped or pureed carrots in toddlers. Previous experience with different textures was the strongest predictor of enjoyment of the chopped carrots and was also associated with amount consumed. This again suggests that amount consumed and eating duration are driven by enjoyment as well as hunger.

As noted previously, Skinner et al. (1998) examined the expression of infant and toddler food preferences alongside other mealtime communication behaviours. They did so using open‐ended questions to explore how mothers identified food preferences in their infants. Mothers identified behaviours such as opening the mouth readily as the spoon approached and consuming a large amount, as indications of liking, while dislike was judged through facial expression and body movements (throwing food and head turning).

## Discussion

**Figure 2:**
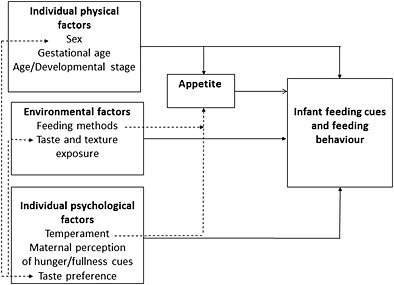
Main influencing factors on feeding behaviour in the first 2 years of life (established connections in solid lines, impact of individual factors on appetite and interactions between factors in broken lines).

The purpose of this review was to consider the evidence regarding infants' feeding cues, along with factors that affect the expression and perception of these. The review revealed that feeding cues and behaviours are shaped by numerous issues. These can be conceptualised in terms of individual psychological factors, infants' physical attributes and environmental factors (Fig. 2). It is important to note, however, that many of these factors are inter‐connected; e.g., psychological factors such as food preference influence consumption but are influenced themselves by environmental factors such as exposure (Mennella et al. 2001; Blossfield et al. 2005) and other individual psychological factors such as temperament (Forestell & Mennella 2012).

### Infant feeding cues and feeding behaviour

Maternal reports indicate that mothers use many cues to assess hunger and satiation (Skinner et al. 1998; Anderson et al. 2001; Hodges et al. 2008). Both general and specific cues may indicate hunger and fullness (Hodges et al. 2008), and there are indications that cues vary in form and intensity and with developmental stage (Hodges et al. 2008; Hodges et al. 2013; Skinner et al. 1998). Notwithstanding insights from maternal reports, this literature is relatively small. In addition, the heterogeneous aims and contexts of different studies confound attempts to draw simple conclusions: Anderson et al. (2001) examined the specific context of weaning, while Hodges et al. (2013) investigated responsive feeding. Studies differ also in the amount of detail provided regarding feeding cues and in the methods used to investigate feeding. Only Skinner et al. (1998) and Hodges et al. (2008) provided detailed information regarding feeding cues and details of developmental aspects of hunger and satiation behaviours. Meanwhile, only Skinner at al. (1998) employed a longitudinal approach, and only Hodges et al. (2013) developed a validated tool for observing hunger and fullness cues (the RCFCS). This, however, is not primarily concerned with tracking cues so much as measuring responsive feeding.

Observational studies of infants in controlled conditions suggest that different motor and sucking behaviours are indicative of hunger and satiation (Lew & Butterworth, 1995; Turkewitz et al. 1966; Paul et al. 1996) and vary with infant age (Paul et al. 1996). Such research provides insights regarding fine details of hunger and satiation behaviours that are less apparent in maternal reports. However, the research in this area has limitations. Again, the literature is small. There are also methodological questions with some papers failing to report issues that may bias results, e.g. observer blindness to experimental condition (Lew & Butterworth 1995; Turkewitz et al. 1966). Only Paul et al. (1996) observed the same infants over an extended period (i.e. months rather than days), and only they compared behaviours before and after feeding with those during feeding.

Alongside the observational work conducted in controlled conditions, Young & Drewett (2000), Parkinson & Drewett (2001) and Van Dijk et al. (2009) conducted observations of normative infant eating behaviour in naturalistic settings. Meanwhile, Reau et al. (1996) investigated normative feeding behaviour using survey methods. It is a relative strength that the feeding norms literature includes both observational and longitudinal enquiry. Furthermore, evidence from these studies is generally consistent regarding ‘gross’ aspects of feeding behaviour such as meal duration, intake and the impact of developmental changes on feeding. They also indicate that behaviours such as food refusal (which might be perceived as satiation) are common, particularly at transition points such as weaning. Studies of infant feeding norms therefore have implications for understanding the contextual parameters of feeding cues in infants. Moreover, in the case of Young & Drewett (2000), they provide insights into the impact of different kinds of food (savoury vs. sweet) on eating behaviour.

### Individual psychological factors

An important indication from several studies is the key role mothers play in interpreting feeding cues. Crucially, this highlights the dyadic nature of feeding interactions. As noted, mothers' interpretation of cues is not based solely on infant behaviour but also infant characteristics and external cues such as time (Anderson et al. 2001; Hodges et al. 2008). Importantly, studies indicate that hunger cues are more salient to mothers than satiation cues (Hodges et al. 2013). The role of mothers in interpreting feeding cues is also evident in associations between maternal characteristics and how feeding cues are perceived. In particular, maternal characteristics such as obesity appear to be associated with lower responsiveness to infant fullness (Hodges et al. 2013).

Evidence suggests that infant temperament may influence feeding behaviour in terms of enjoyment of novel foods or intake of food (Darlington & Wright 2006; Wasser et al. 2011; Forestell & Mennella 2012; McMeekin et al. 2013). Most studies in this area have been concerned with associations between temperament and weight gain or temperament and maternal feeding practices. Several explanations may account for these associations, making it difficult to draw simple conclusions. Darlington and Wright's (2006) finding that infants with high distress to limitations gained weight quickly may be explained in relation to maternal responses to these babies. Infants with high distress to limitations were reported to sleep less and to fuss more, and may have received additional feeds to comfort them. This interpretation is supported by McMeekin et al.'s (2013) finding that mothers of difficult infants were more likely to feed them as a soothing strategy. Alternatively, mothers in Darlington et al.'s (2006) study may have fed demanding babies more as a result of misinterpreting fractiousness as hunger. A further possibility is that this group of infants may simply have been more hungry and demanding because of rapid growth (Darlington & Wright 2006).

Darlington and Wright's (2006) finding that infants with high fearfulness scores showed slower weight gain is harder to explain. The authors suggest such infants may have difficulty expressing their needs, although no evidence is provided for this. The precise mechanisms behind associations between temperament and infant weight therefore remain unclear. The picture is further complicated by findings that maternal characteristics may shape responses to infants with demanding temperaments (Darlington & Wright, 2006; Wasser et al. 2011). While these findings confound attempts to identify causal relationships between infant feeding and infant temperament, they again highlight the bidirectional nature of feeding interactions.

A further difficulty in interpreting the infant temperament and feeding behaviour literature arises from differences in study characteristics (Bergmeier et al. 2014). Different temperament measures were used by McMeekin et al. (2013) from those used by Darlington & Wright (2006) and Wasser et al. (2011) (the STSI and the IBQ, respectively). Furthermore, infants in Darlington's study were younger than those in Wasser et al.'s (2011) and McMeekin's (2013) research (8–12 and 8–72 weeks). In addition, the cross‐sectional nature of much research to date limits how far conclusions can be drawn regarding associations between infant temperament and weight gain.

### The impact of physical characteristics

As discussed, infant age appears to affect how feeding cues are expressed, while Llewellyn et al.'s (2012) twin study provides evidence that appetitive behaviours are also determined in part by genotype. Llewellyn et al.'s (2011) large scale study of infant appetite lends credibility to the idea that characteristics such as sex and birthweight influence appetite and therefore the expression of hunger and satiation. There is additional evidence that characteristics such as sex, birthweight or prematurity influence feeding behaviours and potentially feeding cues (Nisbett & Gurwitz 1970; Hwang, 1978; Wright 1986, Stevenson et al. 1990). Such studies involved direct observation with appropriate procedures taken in relation to this (inter‐rater reliability and observer blindness). This is a relative strength. However, findings from some studies have been brought into question by more recent research. Similar levels of breastmilk consumption by males and females led Wright (1986) to conclude that reports of different hunger cues in male and female infants arose from maternal perceptions rather than infant behaviour. Recent research, though, suggests that the breast milk of mothers of boys is higher in energy than that of mothers of girls (Powe et al. 2010). This casts doubt on assumptions that the breast milk to which male and female infants are exposed is necessarily the same, although it provides some basis for concluding that infant sex might (indirectly) play a role in the expression of hunger. The studies by Nisbett & Gurwitz (1970) and Hwang (1978) provide additional evidence that infant sex may shape aspects of feeding behaviour (response to taste or sucking behaviour). However, the lack of homogeneity in studies relating to sex and feeding behaviour hampers attempts to draw straightforward conclusions.

While Wright's (1986) conclusions regarding sex have been challenged by recent research, the same cannot be said of studies of prematurity and later feeding behaviour. The literature search generated very little research on the impact of prematurity on feeding in infancy beyond the first days and weeks of life. However, findings from Stevenson et al. (1990) and Llewellyn et al. (2011) suggest that premature babies may exhibit different feeding cues or different appetitive behaviours at 8 months of age and beyond. This merits further investigation particularly given that this may impact on mothers' feeding responses; i.e., mothers of pre‐term infants may capitalise on open mouth postures during vocalisation as opportunities to feed (Stevenson et al. 1990).

### Environmental factors

Like studies of infant feeding norms, the research regarding the impact of feeding method on feeding behaviour provides contextual information for understanding hunger and satiation in infancy. Ventura et al.'s (2012) findings that formula milk composition affects speed of satiation and length of satiety have implications for the frequency with which hunger cues are observed and the speed with which these abate. Meanwhile, Wright et al.'s (1980) finding of differences in consumption and temporal feeding patterns between breastfed and formula‐fed infants may have implications for interpreting infant hunger and satiation; the authors suggest that a lack of variation in the parameters of formula feeds compared with breast feeds may make it harder for mothers to interpret hunger and satiation in formula‐fed infants.

As noted, environmental factors such as exposure to different food characteristics give rise to individual psychological factors by influencing food preferences. More importantly for this review, however, the literature indicates that consumption and duration of feeding are both associated with liking, while cessation of feeding is associated with dislike. This has been reported across several food characteristics—taste, flavour and texture (Mennella et al. 2001; Mennella et al. 2009; Blossfield et al. 2005). Such findings are significant for understanding feeding cues as intake and continued feeding are perceived as hunger in mothers' reports (Anderson et al. 2001). Similarly, cessation of eating is perceived to indicate satiation (Gross et al. 2010; Hodges at al. 2008). The question is therefore whether cues associated with liking and dislike can be differentiated from those associated with hunger/satiation. Clearly, this has implications for mothers deciding when a child has eaten enough.

Facial expression appears to provide some basis for differentiating between dislike and satiation as negative expressions appear to indicate dislike (Skinner et al. 1998; Mennella et al. 2001; Blossfield et al. 2005, Forestell & Mennella, 2012). Distinguishing between eating driven by liking rather hunger, however, is more challenging. Studies provide few clues regarding liking cues beyond facial relaxation and smiling (Skinner et al. 1998; Mennella et al. 2009). Furthermore, what is not known is the relative contribution made by hunger and hedonic aspects of eating to issues such as consumption and duration of eating.

### Review limitations

While the review has explored a large amount of research regarding hunger and satiation in infancy, it has limitations. Only published papers were considered, and a search of the grey literature was not performed; important findings may therefore have been omitted.

A second limitation lies in the heterogeneity of the studies discussed. While the diverse nature of the papers reviewed might be considered a strength, this presents challenges when synthesising findings and drawing conclusions. The varying topics and methods of investigation involved in the reviewed papers make comparison difficult, even for studies within the same area of enquiry.

Finally, while studies with the lowest ratings were excluded from the review, it is noted that the quality of some remaining studies is relatively low. There were also some discrepancies between raters on quality for a small number of papers, but because inter‐rater agreement over all was high, no further action was taken. Such limitations therefore should be taken into account when considering the findings of the review.

### Review implications

This review has identified several gaps in the literature regarding infant feeding cues. In particular, there is a lack of observational research in naturalistic settings to corroborate maternal descriptions of feeding cues. Such research would facilitate assessment of the respective impacts of infant and maternal characteristics on how cues are perceived. Meanwhile, much research in this area has been cross‐sectional, and new, longitudinal studies are needed. These would elucidate the impact of developmental issues on feeding and provide insights for mothers trying to decipher cues in the context of changing infant behaviours. Furthermore, in order for the aforementioned recommendations to be carried out there is a need for researchers to develop validated tools for observing infant hunger and satiation.

Some observational research regarding infant hunger and satiation has been conducted under controlled (rather than naturalistic) conditions. However, the lack of homogeneity in this work along with methodological limitations of some studies means that higher quality, more homogenous research is required. In addition, studies to date have examined behaviour before and after feeding rather than within a feeding episode. This represents a gap in our knowledge, as it is within the mealtime context itself that mothers have to assess and respond to infants' satiation. Regarding infant characteristics and hunger and satiation, the review has identified gaps in the research in terms of associations between temperaments and feeding cues. Research using consistent measures is needed to facilitate comparisons across studies. Again, the lack of observational research in the area is problematic. Addressing this would elucidate the precise contributions of infant and maternal characteristics to reported associations between infant temperament and weight. Such research could inform responsive feeding interventions, should it confirm that infants with difficult temperaments are at risk of being over‐fed.

Regarding broad conclusions that can be drawn about infants' physical attributes and feeding cues, it seems that a range of characteristics (gestational age at birth, birthweight and sex) may shape feeding behaviour. However, the relative impact of different characteristics is difficult to judge as a disparate range of behaviours has been studied (e.g. response to taste, sucking behaviour and fussiness during meals). A more coherent programme of research is indicated to investigate the impact of different infant characteristics on the same aspects of feeding.

A final area for further enquiry indicated by the review concerns the need to understand the contributions that hunger/satiation and liking/dislike make to infant intake of food. Additional studies to examine feeding behaviour in the contexts of main and sweet courses are needed. Likewise, research to determine how infants communicate liking of food is needed given that studies so far provide few clues regarding cues associated with liking. This has implications for healthy eating initiatives given evidence that infant consumption is not only driven by hunger.

In summary, the existing literature provides insights into many aspects of hunger and satiation in infancy; however, there are significant gaps in our knowledge. Addressing these would make a valuable contribution to our understanding of infant feeding cues and what infants bring to feeding interactions with parents. This is particularly important given the implication of maternal feeding practices in the development of infant obesity risk.

## Source of funding

JM is funded by an ESRC/WRDTC PhD award.

## Conflicts of interest

The authors declare that they have no conflicts of interest.

## Contributions

JM designed the research, undertook the literature search and drafted the review; JM, MH, SHJ, SC, HW and CV advised on search strategy and carried out quality ratings on reviewed papers; JM developed the tables and Fig. 1. SC developed Fig. 2. JM wrote the paper and re‐drafted this in response to comments from MH, SHJ, SC, HW and CV. All authors read and approved the final paper.

## Supporting information

Supporting info item
