# Supplementary material for: Communicating hunger and satiation in the first 2 years of life: a systematic review
Source: Matern Child Nutr. 2015 Dec 1;12(2):205–28. doi: 10.1111/mcn.12230 (PMC4991302; doi:10.1111/mcn.12230)
Supplement: Supplementary file 1 — Supporting info item [file MCN-12-205-s001.docx]

**Communicating hunger and satiation in the first two years of life: a systematic review – supplementary materials.**

**Supplementary table - Mapping of ‘Infant’ and ‘Feeding’ to subject headings in Medline, Maternal and Infant Health and PsycINFO databases.**

| Medline  Infant Infant Food/ or Infant Behavior/ or Infant Formula/ or exp Infant/ or Infant, Newborn/Maternal and Infant Health.  Feeding Breast Feeding/ or Feeding Methods/ or Formula Feeding/ or Feeding Behavior/ |
| --- |
| PsycINFO  Infantexp Infant Development/  Feedingexp Formula Feeding/ or exp Breast Feeding/exp Eating Behavior/ |
| Maternal and Infant Health  Infant Infant – premature/ or Infant - newborn  Feeding Breastfeeding/ or Infant feeding/Formula feeding |

**Supplementary table - Infant hunger and satiation cues identified from maternal perception studies.**

|  | Anderson et al. (2001) | | Gross et al.(2010) | Hodges et al. (2008) | Hodges et al. (2013) | Skinner et al. (1998) | Wright(1986) |
| --- | --- | --- | --- | --- | --- | --- | --- |
| INFANT AGE | **8-18 weeks** | **0-5 months** | | **3-12 months** | **7-24 months** | **2-24 months** | **0-2 months** |
| Hunger cues | ‘Hungry cry’, interest in others’ food, responsiveness to food smells, looking for more milk, sucking an empty bottle, feeding more frequently, feeding ‘furiously’, chewing hands, changed sleep pattern, being unsatisfied by feed, excitement at seeing solid food. | Crying  Hand sucking | | Crying/fussing, rooting, sucking or mouthing, licking, lips, squirming, negative affect, reaching for or grabbing food, staring, pointing at food, ‘non-verbal communication’, keeping quiet, going to refrigerator, taking bottle from bag. | Increased alertness, hand sucking, leaning towards food, excitatory limb movements, crying, mouthing, reaching for food. | Opening mouth when spoon approaches/when hungry, crying, reaching for spoon, leaning forward as spoon approaches, canonical babble to show readiness for next spoonful, enjoying eating during first half of meal/eating readily | Unsettled baby, increased frequency of feeds, mouthing, whimpering for food, agitation, crying. |
| Satiation cues | Contentment  Heavy breathing  Being satisfied  Wishing to eat less often. | Turning head away from breast or bottle  Baby ‘knowing’ when s/he is full. | | Detaching from nipple, refusing food, spitting food out, closing mouth, turning away, sleeping, placing hand to mouth/touching mouth or head, head shaking, playing with food, slowed pace of eating, crying or fussing, throwing food, saying ‘no’, distractibility, stomach distension, trying to escape. | Not directly described in study | Looking distressed, turning head away, crying as feeding continues, closing mouth, losing interest in eating, losing interest after first one or two bites. | Not reported. |

**Supplementary table - Findings from observational studies of infant behaviours before, during and after feeding**

| Lew and Butterworth (1995) | Turkewitz et al. (1996) | | Paul et al. (1996) | |  | |
| --- | --- | --- | --- | --- | --- | --- |
| Infant Age Newborn | Newborn | | 2-26 weeks | |  | |
| Prior to Feeding More hand-mouth contacts  preceded by open mouth  postures are observed. | | Greater proportion of  hand/finger flexions  observed prior to feeding | | Increased motor activity  from 18+ weeks of age | |  |
| During  Feeding |  | | Fast sucking movements at the start of feeds (at all ages) followed by pauses. Motor activities decrease in frequency during feeding in all ages. | |  | |
| After  Feeding | Lower proportion of  hand/finger flexions  observed after feeding | | Decreased muscle tone in two week old infants. Increased motor activity from 18 weeks of age | |  | |
